# Chemical Genetics Reveals a Specific Requirement for Cdk2 Activity in the DNA Damage Response and Identifies Nbs1 as a Cdk2 Substrate in Human Cells

**DOI:** 10.1371/journal.pgen.1002935

**Published:** 2012-08-23

**Authors:** Lara Wohlbold, Karl A. Merrick, Saurav De, Ramon Amat, Jun Hyun Kim, Stéphane Larochelle, Jasmina J. Allen, Chao Zhang, Kevan M. Shokat, John H. J. Petrini, Robert P. Fisher

**Affiliations:** 1 Department of Structural and Chemical Biology, Mount Sinai School of Medicine, New York, New York, United States of America; 2 Program in Biochemistry and Program in Cell and Molecular Biology, Weill Cornell Graduate School of Medical Sciences, New York, New York, United States of America; 3 Molecular Biology Program, Memorial Sloan-Kettering Cancer Center, New York, New York, United States of America; 4 Department of Cellular and Molecular Pharmacology, University of California San Francisco, San Francisco, California, United States of America; University of Washington, United States of America

## Abstract

The cyclin-dependent kinases (CDKs) that promote cell-cycle progression are targets for negative regulation by signals from damaged or unreplicated DNA, but also play active roles in response to DNA lesions. The requirement for activity in the face of DNA damage implies that there are mechanisms to insulate certain CDKs from checkpoint inhibition. It remains difficult, however, to assign precise functions to specific CDKs in protecting genomic integrity. In mammals, Cdk2 is active throughout S and G2 phases, but Cdk2 protein is dispensable for survival, owing to compensation by other CDKs. That plasticity obscured a requirement for Cdk2 activity in proliferation of human cells, which we uncovered by replacement of wild-type Cdk2 with a mutant version sensitized to inhibition by bulky adenine analogs. Here we show that transient, selective inhibition of analog-sensitive (AS) Cdk2 after exposure to ionizing radiation (IR) enhances cell-killing. In extracts supplemented with an ATP analog used preferentially by AS kinases, Cdk2^as^ phosphorylated the Nijmegen Breakage Syndrome gene product Nbs1—a component of the conserved Mre11-Rad50-Nbs1 complex required for normal DNA damage repair and checkpoint signaling—dependent on a consensus CDK recognition site at Ser432. In vivo, selective inhibition of Cdk2 delayed and diminished Nbs1-Ser432 phosphorylation during S phase, and mutation of Ser432 to Ala or Asp increased IR–sensitivity. Therefore, by chemical genetics, we uncovered both a non-redundant requirement for Cdk2 activity in response to DNA damage and a specific target of Cdk2 within the DNA repair machinery.

## Author Summary

Multiple cyclin-dependent kinases (CDKs) control human cell proliferation, but it remains unclear how functions of different CDKs are coordinated during unperturbed cell division or after dividing cells incur DNA damage. DNA lesions activate checkpoint signaling pathways to inhibit CDK activity, arrest the cell division cycle, and thus prevent loss of genetic information; but an effective response to damage also requires CDK activity to modify components of repair and checkpoint pathways. We took a chemical-genetic approach to ask if a specific CDK, Cdk2, played a specialized, non-redundant role in protecting genomic integrity of human cells. By sensitizing Cdk2 to chemical inhibition, we were able to detect a specific requirement for its catalytic activity in survival of cells after exposure to ionizing radiation (IR). We identified Nbs1, product of the gene mutated in the cancer-predisposing Nijmegen Breakage Syndrome, as a Cdk2 substrate and showed that mutant forms of Nbs1 that cannot be modified by Cdk2 are defective in protecting cells from death due to IR–induced DNA damage. Therefore, our work defines a DNA damage response pathway that depends on catalytic activity of a specific CDK in human cells and suggests a mechanism to promote efficient repair without triggering inappropriate cell division.

## Introduction

In eukaryotes, responses to DNA damage or replication errors must be coordinated with cell division. For example, checkpoint pathways signal the presence of DNA lesions to the cell-cycle machinery, leading to reversible arrest or apoptosis. In reciprocal fashion, the cyclin-dependent kinases (CDKs) that regulate cell-cycle progression also appear to control aspects of the DNA damage response. For example, CDK activity promotes repair of DNA double-strand breaks (DSBs) by homologous recombination (HR) in yeast [1]–[3]. In *Saccharomyces cerevisiae*, CDK phosphorylates the conserved repair protein Sae2 to promote DNA-end resection and thereby channel DSBs into the HR pathway [4]. In metazoans, CDKs have been shown to phosphorylate DNA repair and checkpoint proteins (reviewed in [5], [6]), but the consequences of most of those phosphorylations remain unclear. One exception is the Sae2 homolog CtIP, the phosphorylation of which appears to facilitate resection, and might couple DSB repair pathway choice to cell-cycle position in mammalian cells [7], [8].

It is also uncertain *which* CDK/cyclin complexes regulate responses to DNA damage in metazoans. In yeast, a single CDK catalytic subunit triggers entry to both S phase and mitosis, whereas metazoans normally rely on multiple CDKs [9]. The latter arrangement suggests a potential solution to the problem of maintaining some CDK activity in the face of inhibitory checkpoint signals: specialization of individual CDKs to evade those signals. In mammalian cells, Cdk2 is the nearly exclusive partner of cyclin E, which is expressed near the G1/S boundary, and the preferred partner of cyclin A early in S phase. Later in S phase, cyclin A begins binding Cdk1 [10], to trigger initiation from late-replicating origins [11] and attenuate S phase-specific gene expression [12]. Finally, Cdk1 assembles with cyclin B during S and G2 phases, and is activated late in G2 to promote mitosis. Despite the temporal restriction and apparent functional specialization of CDKs in mammalian cells, discerning non-redundant functions of specific catalytic subunits has been difficult. Cells lacking Cdk2 can divide more or less normally, and *Cdk2^−/−^* mice are viable, but infertile due to a defect in meiosis [13], [14]. Moreover, cells lacking all interphase-specific CDKs can proliferate, albeit more slowly than wild-type cells, by substituting Cdk1 for the “correct” partners in complexes with cyclins D, E and A [15].

Because of that plasticity, eliminating or reducing expression of individual CDKs by gene disruption or RNA interference (RNAi) may not reveal which functions those CDKs perform, perhaps exclusively, when they are present. For example, Cdk2 is likely to control the onset of DNA replication, based on its activation timing [10], [11] and the lack of a Cdk1 requirement for S-phase entry when Cdk2 is present [16]. By the same logic, Cdk2 might take a lead role in influencing the choice of DSB repair pathway early in S phase. Consistent with that idea, loss or depletion of Cdk2 was reported to increase radiation-sensitivity and cause defects in DNA damage repair and checkpoint signaling [17]–[19]. It was later suggested, however, that the requirement for CDK activity in response to DNA damage is a general one, with no specific need for Cdk2 either to repair damage or to resume the cell cycle after repair is complete [20]. More recently, an exclusive function was ascribed to Cdk2 in imposing a G2/M checkpoint arrest in cells lacking wild-type function of the tumor suppressor p53 [21].

Precise temporal control over the activity of individual CDKs, which was needed to uncover the Cdk1 requirement in yeast DSB repair [1], [3], has not heretofore been possible in metazoans; none of the available small-molecule inhibitors can discriminate between Cdk1 and Cdk2, and depletion of specific CDKs allows ectopic CDK-cyclin pairs to take over functions normally performed by the missing enzyme [15], [22], [23]. To dissect the precise roles of different CDKs in human cells, we took a chemical-genetic approach, in which a bulky amino acid residue in the active site—the gatekeeper—is mutated to Gly, creating extra space to accommodate bulky adenine analogs [24]. Analog-sensitive (AS) Cdk2 is susceptible to inhibition by non-hydrolyzable analogs that bind poorly to wild-type kinases, and able to use bulky ATP analogs (as well as natural ATP) as substrates [25], [26]. By a gene targeting strategy we applied previously to human Cdk7 [27], we replaced both wild-type copies of *Cdk2* with *Cdk2^as^* in human cells, and uncovered a requirement for Cdk2 activity in cell proliferation, which was missed by gene knockout- and RNAi-based studies [28].

Requirements for Cdk2 activity in the DNA damage response are also likely to have escaped detection. Here we show that transient treatment with an allele-specific inhibitor decreased survival of *Cdk2^as/as^* but not *Cdk2^+/+^* cells after exposure to ionizing radiation (IR), indicating a specific requirement for Cdk2 activity in orchestrating an effective DNA damage response. In whole-cell extracts, Cdk2^as^ labeled Nbs1, product of the gene mutated in the autosomal recessive Nijmegen Breakage Syndrome (NBS) of microcephaly, immunodeficiency, and increased incidence of hematopoietic malignancy (reviewed in [29]). Nbs1 is part of the essential Mre11-Rad50-Nbs1 complex, which functions in recognition and repair of DSBs (reviewed in [30]). We mapped the Cdk2-mediated phosphorylation of Nbs1 to Ser432. In vivo, that phosphorylation occurs during S phase after the Mre11 complex is recruited to chromatin, and is prevented by general CDK blockade or delayed and diminished by specific inhibition of Cdk2. Mutations of Nbs1-Ser432 that prevent phosphorylation increase sensitivity to cell-killing by IR, and thus phenocopy selective inhibition of Cdk2. Therefore, by chemical genetics we have uncovered a specifically Cdk2-dependent pathway within the DNA damage response of human cells.

## Results

### Cdk2 activity is required for normal IR–resistance

As previously reported [28], the Phe80-to-Gly gatekeeper mutation, which rendered Cdk2 AS, also impaired binding to cyclin A in vivo, but this defect was corrected by treating cells with bulky adenine analogs. When normal CDK-cyclin pairing was thus maintained, sustained treatment with 1-(*tert*-Butyl)-3-(3-methylbenzyl)-1H-pyrazolo[3,4-d]pyrimidin-4 amine (3-MB-PP1)—a selective inhibitor of AS, but not wild-type, CDKs—impaired proliferation of asynchronously growing *Cdk2^as/as^* cells. Acute inactivation of Cdk2, in synchronized populations of untransformed, human telomerase-expressing retinal pigment epithelial (RPE-hTERT) cells, impeded passage through the G1 restriction point (when continued cell-cycle progression becomes independent of mitogen stimulation [31]) and entry into S phase, and increased the frequency of arrest in the G0 or G1 phase of the cell cycle [28].

**Figure 1:**
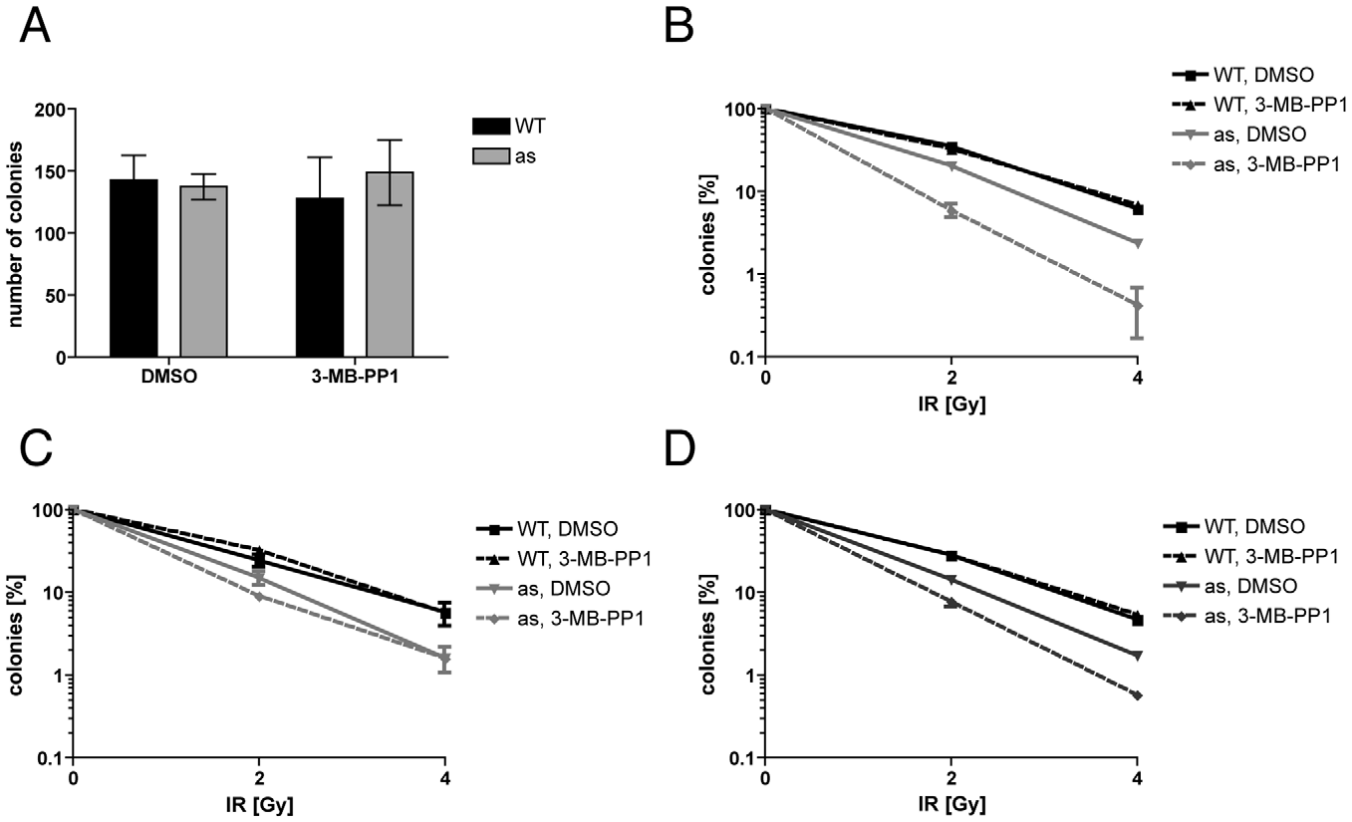
Cdk2 activity is required for normal DNA damage resistance. (A) Transient treatment with 10 µM 3-MB-PP1 for 48 hr does not affect efficiency of colony formation by wild-type or *Cdk2^as/as^* RPE-hTERT cells. (B) Treatment with 10 µM 3-MB-PP1 before and after irradiation (24 hr each) sensitizes *Cdk2^as/as^* RPE-hTERT cells to killing by IR. (C) Pre-treatment with 3-MB-PP1 for 24 hr does not sensitize *Cdk2^as/as^* cells to IR. (D) Treatment with 3-MB-PP1 for 24 hr after IR sensitizes *Cdk2^as/as^* cells to killing. Error bars are +/− standard deviation (SD) of duplicates.

The effects of Cdk2 inhibition on cell proliferation were reversible; a 48-hr treatment of asynchronous *Cdk2^as/as^* RPE-hTERT cells with 10 µM 3-MB-PP1, followed by removal of the drug, allowed colony formation with no loss of efficiency (Figure 1A). We were therefore able to test the effect of transient Cdk2 inhibition on survival after IR-induced DNA damage. *Cdk2^as/as^* or wild-type cells were pre-treated with DMSO or 3-MB-PP1 for 24 hr; γ-irradiated with 2 or 4 Gy, re-plated and incubated for an additional 24 hr in the same condition as the pre-treatment; then washed and returned to fresh, drug-free medium for 14 d before colonies were counted. Even in the absence of the drug, the mutant had increased sensitivity to IR compared to wild-type cells (Figure 1B), suggesting that *Cdk2^as^* is hypomorphic. This could be due to the cyclin-binding defect and incomplete compensation by other CDKs, which would be consistent with results in *Cdk2^−/−^* cells [19]. Transient treatment with 10 µM 3-MB-PP1 further decreased survival by *Cdk2^as/as^* cells ∼10-fold, but did not affect sensitivity of wild-type cells, indicating that Cdk2 activity is specifically required for resistance to DNA damage caused by IR.

In the preceding experiment, Cdk2 inhibition was implemented 24 hr before, and maintained for 24 hr after, exposure to IR. Enhanced cell-killing by this regimen could indicate a requirement for Cdk2 to phosphorylate repair or checkpoint effectors, or some cell-cycle derangement occurring during the pre-treatment period. To distinguish between these mechanisms, we varied the relative timing of Cdk2 inhibition and irradiation. *Cdk2^as/as^* cells treated with 3-MB-PP1 for 24 hr prior to irradiation and return to drug-free medium were barely sensitized to IR, compared to mock-treated cells (Figure 1C). In contrast, a 24-hr 3-MB-PP1 treatment initiated at the time of irradiation increased IR-sensitivity, relative to mock treatment, to nearly the same extent as did the 48-hr, “before-and-after” exposure (Figure 1D). Therefore, Cdk2 catalytic activity is specifically required after DNA damage occurs to promote survival of human cells exposed to IR.

### Cdk2 phosphorylates Nbs1

**Figure 2:**
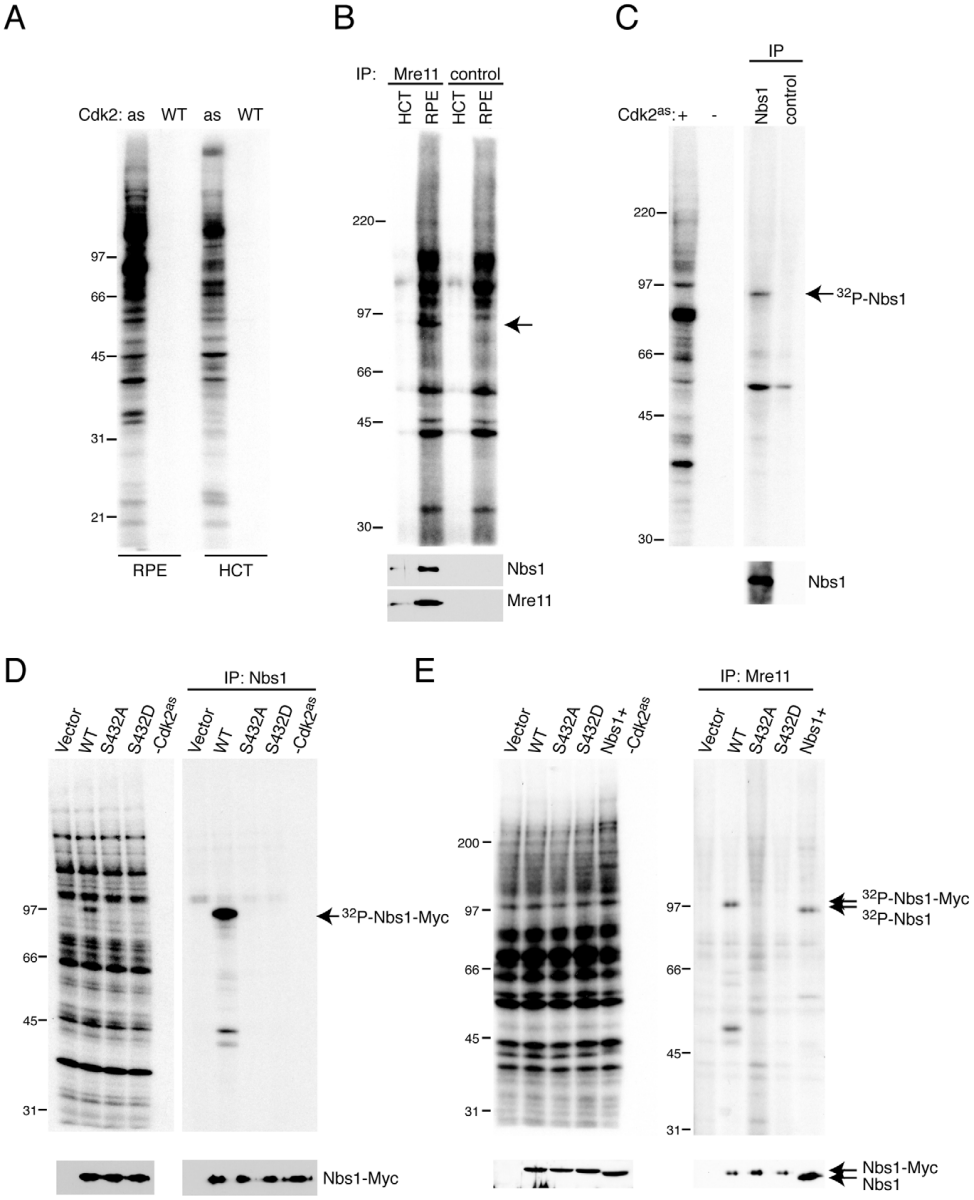
Nbs1 is phosphorylated by Cdk2 on Ser432 in human whole-cell extracts. (A) Labeling by Cdk2^as^/or Cdk2^WT^/cyclin A, as indicated, with [γ-^32^P]*N6*-(benzyl)-ATP in RPE-hTERT or HCT116 whole cell extracts. (B) Anti-Mre11 immunoprecipitates from labeling reactions analyzed by autoradiography (top) and anti-Nbs1 and -Mre11 immunoblot (bottom). Arrow indicates band at position of Nbs1, which did not appear in mock immunoprecipitates lacking antibody (“control”). (C) Anti-Nbs1 immunoprecipitates from labeling reactions analyzed by autoradiography (top) and anti-Nbs1 immunoblot (bottom). (D) Reticulocyte lysates programmed with indicated cDNAs were labeled by Cdk2^as^. Labeling in extract (left) and anti-Nbs1 immunoprecipitates (right) was detected by autoradiography, and expression of Nbs1 isoforms confirmed by immunoblot (bottom). (E) NBS-T cells were transiently transfected with empty vector or ones encoding Myc-tagged Nbs1 variants, as indicated. Labeling by Cdk2^as^ was detected by autoradiography of extract (left) and anti-Mre11 immunoprecipitates (right), and equal expression and recovery of Nbs1 isoforms were confirmed by immunoblot (bottom). Nbs1+ denotes control cells expressing full-length Nbs1 endogenously.

To identify targets through which Cdk2 executes its functions in DNA damage responses, we labeled proteins in whole-cell extracts of wild-type RPE-hTERT and HCT116 human colon carcinoma cells with purified Cdk2^as^/cyclin A and the analog substrate [γ-^32^P]*N6*-(benzyl)-ATP [32], [33]. Proliferation of both cell types is sensitive to selective inhibition of Cdk2 [28]. RPE-hTERT cells are largely intact in their responses to DNA damage [34], however, whereas HCT116 cells are defective in mismatch repair and can be deficient in Mre11-complex function [35]. Although overall labeling patterns were similar, several bands were more heavily phosphorylated in RPE-hTERT, as opposed to HCT116, extracts—including prominent signals at >100, ∼80 and ∼35 kDa (Figure 2A). We observed similar, cell type-specific labeling in extracts of the two lines expressing Cdk2^as^ endogenously [28]. There was no labeling, however, when wild-type Cdk2 complexes were substituted for Cdk2^as^, indicating that all visible signals represent direct targets of Cdk2 activity.

To test if Cdk2^as^ phosphorylated the Mre11 complex, we subjected labeled extracts to anti-Mre11 immunoprecipitation (Figure 2B). Although we did not recover the ∼80 kDa species that approximately matches Mre11 in electrophoretic mobility (and remains unidentified), a labeled polypeptide the size of Nbs1 (∼95 kDa) co-precipitated with Mre11. A labeled band of similar mobility appeared in anti-Nbs1 immunoprecipitates (Figure 2C), confirming that Cdk2^as^ selectively phosphorylates Nbs1 in crude extracts. Labeling intensity correlated with Nbs1 abundance in different cell types (Figure S1).

Human Nbs1 contains one match, at Ser432, to the consensus CDK recognition motif S/T-P-X-K/R (where X is any residue). This site is conserved in metazoans, and both fission yeast Nbs1 and Xrs2—the corresponding subunit of the budding yeast Mre11 complex—contain potential CDK phosphorylation sites [36]–[38]. To map phosphorylation(s) by Cdk2 in human Nbs1, we transcribed and translated cDNAs encoding the wild-type protein (WT) and mutants with Ser432 changed to Ala (S432A) or Asp (S432D) in reticulocyte lysates, which were then labeled with Cdk2^as^/cyclin A and [γ-^32^P]*N6*-(benzyl)-ATP. Whereas all three forms of Nbs1 were translated efficiently, only the wild-type version was labeled (Figure 2D). Next, to ask if Cdk2 phosphorylated Nbs1 in the Mre11 complex with the same dependence on Ser432, we performed labeling in extracts of NBS-T cells—transformed fibroblasts derived from a patient with NBS, which do not express full-length Nbs1—or the same cells expressing full-length Nbs1 after transient transfection. Wild-type, S432A and S432D Nbs1 variants were expressed at equal levels and recovered with similar efficiency in anti-Mre11 immunoprecipitates. Only the wild-type protein was labeled, however (Figure 2E), confirming that an intact Ser432 residue is required for Cdk2^as^ to phosphorylate Nbs1 within the Mre11 complex.

### Cell-cycle CDKs phosphorylate Nbs1-Ser432 in vitro and in vivo

**Figure 3:**
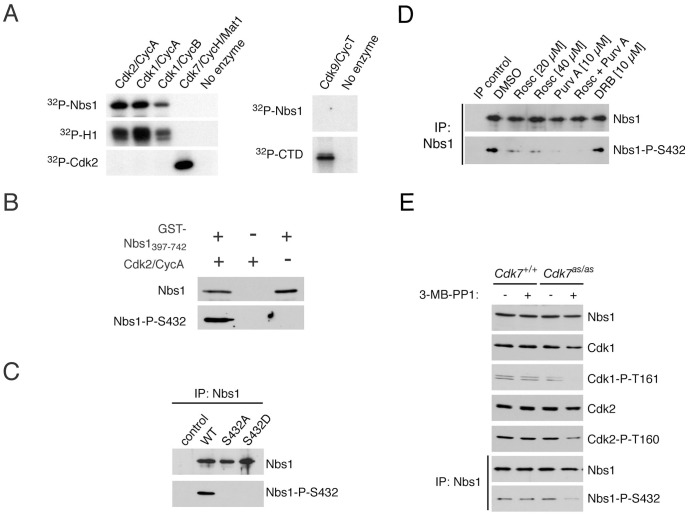
CDKs phosphorylate Nbs1 on Ser432 in vitro and in vivo. (A) Purified recombinant Nbs1 fragment (GST-Nbs1(397-742)) was incubated in vitro with indicated CDK/cyclin complexes and [γ-^32^P]-ATP. Histone H1, monomeric Cdk2, and the carboxyl-terminal domain (CTD) of RNA polymerase II, served as control substrates for Cdk1 or -2, Cdk7, and Cdk9, respectively. (B) Incubation of GST-Nbs1(397-742) in vitro with Cdk2/cyclin A, Mg^++^ and ATP generates an epitope recognized by the anti-Nbs1-S432-P antibody in immunoblots. (C) Wild-type but not Ser432-mutant Nbs1, transiently expressed in NBS-T cells, is recognized by anti-Nbs1-S432-P antibody. (D) Roscovitine and/or purvalanol A treatment of HCT116 cells for 15 hr diminishes Nbs1-Ser432 phosphorylation without affecting Nbs1 levels, whereas DRB has no effect. (E) Treatment of *Cdk7^as/as^* but not wild-type HCT116 cells with 2 µM 3-MB-PP1 for 24 hr decreases phosphorylation of Cdk1 (P-T161), Cdk2 (P-T160), and Nbs1-Ser432.

To ask which human CDK/cyclin pair(s) could phosphorylate Nbs1, we incubated a fragment of human Nbs1 fused to glutathione-*S*-transferase [GST-Nbs1(397-742)] with various purified CDKs (Figure 3A). Cdk2/cyclin A, Cdk1/cyclin A and Cdk1/cyclin B phosphorylated GST-Nbs1(397-742) with similar efficiencies relative to a known, common substrate, histone H1. In contrast, neither Cdk7 nor Cdk9 could label Nbs1 above background levels, although both were active towards their known substrates. We conclude that Nbs1 is a substrate of the cell-cycle effectors Cdk1 and Cdk2.

Antibodies raised against a phospho-Ser432-containing peptide recognized GST-Nbs1(397-742) purified from bacteria only after it was incubated with Cdk2/cyclin A, Mg^2+^ and ATP (Figure 3B). We detected no signal in the Cdk2/cyclin A preparation or when GST-Nbs1(397-742) was incubated in the absence of CDK, confirming specificity of the antibody for phosphorylated Nbs1. The anti-phospho-Ser432 antibody recognized wild-type Nbs1, but not the S432A or S432D variant, expressed in NBS-T cells (Figure 3C), indicating that Nbs1-Ser432 is phosphorylated in vivo.

To ask if CDKs are responsible for Nbs1 phosphorylation in vivo, we treated HCT116 cells with kinase inhibitors. Roscovitine and purvalanol A, which inhibit multiple CDKs, including Cdk1 and Cdk2 [39], [40], diminished phospho-Ser432 without affecting total Nbs1 levels, whereas 5,6-dichloro-1-β-D-ribofuranosyl-benzimidazole (DRB), a transcriptional poison thought to work through inhibition of Cdk9 [41], had no effect (Figure 3D). Because none of these compounds is specific for a single class of kinase, we asked if selective inhibition of Cdk7—the upstream activator of both Cdk1 and Cdk2 [27]—prevented Nbs1 phosphorylation. Treatment with 3-MB-PP1 diminished activating phosphorylation of Cdk1 and Cdk2 in *Cdk7^as/as^* but not wild-type HCT116 cells, and also decreased Nbs1-Ser432 phosphorylation (Figure 3E). Cdk7 is incapable of modifying Nbs1 directly (Figure 3A); a dependence on Cdk7 activity therefore suggests that Nbs1-Ser432 is a target of a CDK (or CDKs) downstream of Cdk7, such as Cdk1 or Cdk2.

### Phosphorylation of Nbs1-Ser432 is cell-cycle regulated

**Figure 4:**
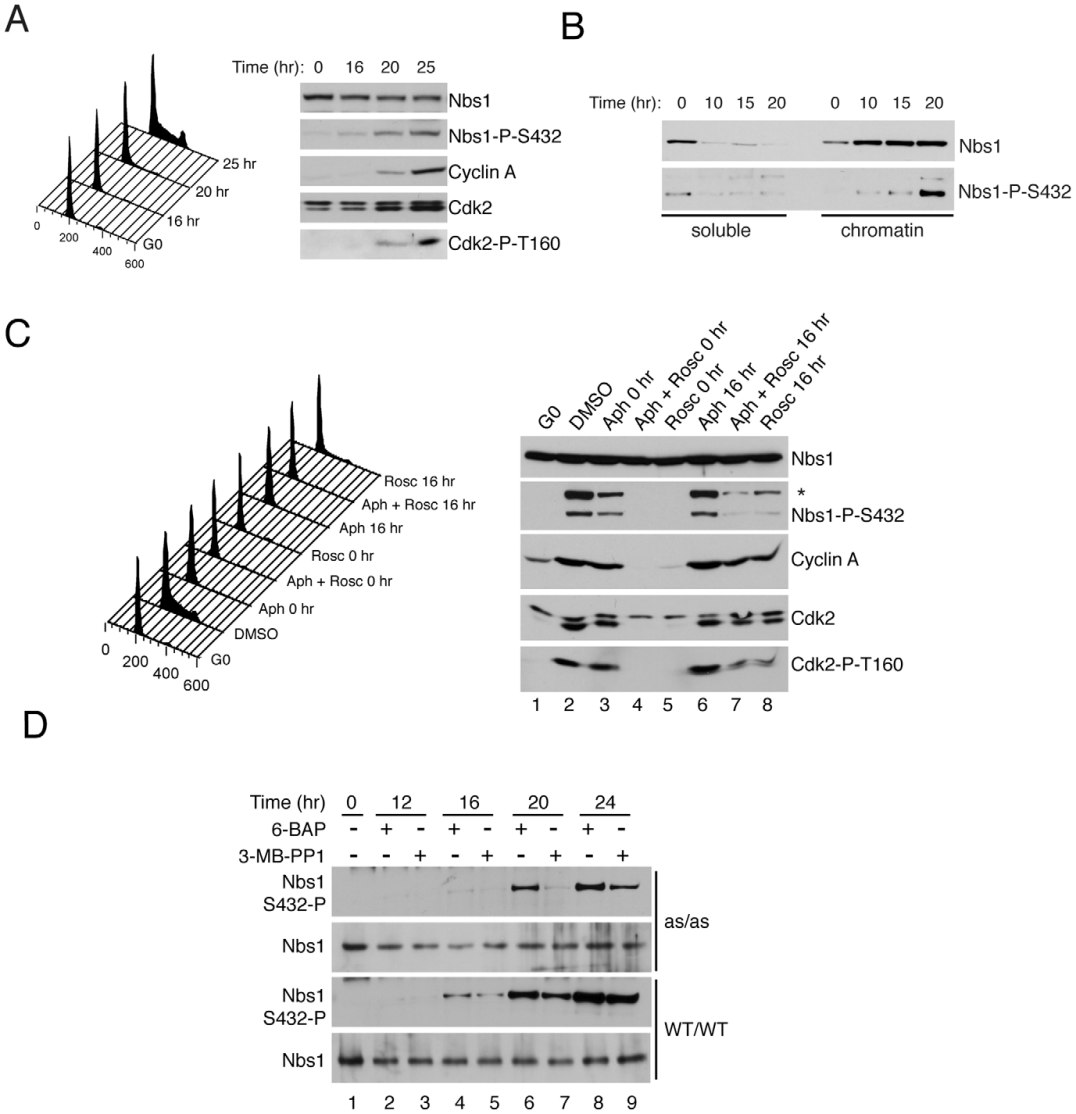
Ser432 is phosphorylated in S phase after Nbs1 recruitment to chromatin. (A) Time course of release from contact inhibition (G0) of RPE-hTERT cells, monitored by flow cytometry for DNA content and immunoblotting for accumulation of cyclin A, activated (Thr160-phosphorylated) Cdk2 and Ser432-phosphorylated Nbs1. (B) RPE-hTERT cells were harvested and fractionated at indicated times after release from G0. Recruitment of Nbs1 to chromatin precedes phosphorylation on Ser432. (C) RPE-hTERT cells treated with DMSO, 4 µg/ml aphidicolin or 20 µM roscovitine at indicated times after release from G0 were monitored for DNA content (*left*) and accumulation of cyclin A, activated Cdk2 and phosphorylated Nbs1 (*right*). Asterisk denotes anti-phospho-Ser432 cross-reactive ∼110 kDa band that is absent in G0 and roscovitine-sensitive, but unlikely to be an *Nbs1* gene product, because it is present in NBS-T cells (Figure S2B) and absent in Nbs1 immunoprecipitates (Figure 3D, 3E). (D) *Cdk2^as/as^* or wild-type RPE-hTERT cells were released from G0 for indicated times in the presence of 0.5 µM 6-BAP or 10 µM 3-MB-PP1, as indicated, and tested for total (top) and Ser432-phosphorylated Nbs1 (bottom).

If Nbs1-Ser432 were a target of Cdk1 and/or Cdk2 in vivo, we would expect its phosphorylation state to fluctuate with cell-cycle position. We tested this prediction in RPE-hTERT cells, which arrest in a quiescent, G0-like state upon growth to confluence, and re-enter the cell cycle synchronously upon re-plating at lower density. Nbs1-Ser432 phosphorylation was low in G0 and increased 20–25 hr after release, concomitant with increases in cyclin A accumulation and activating phosphorylation of Cdk2 (Figure 4A, right), and the appearance of a cell population with >2N DNA content (Figure 4A, left).

The Mre11 complex is recruited to chromatin throughout [42], and required for successful completion of, S phase [43], [44]. To ask if Ser432 phosphorylation correlated with relocalization of Nbs1 to chromatin, we separated extracts of RPE-hTERT cells, harvested at different times after release from G0, into soluble and chromatin-bound fractions. Nbs1 was predominantly associated with chromatin by 10 hr after release, and Ser432 phosphorylation of chromatin-bound Nbs1 increased between 10 and 20 hr (Figure 4B). Treating cells with roscovitine, either at the time of re-plating or 16 hr later, prevented increases in cellular DNA content (Figure 4C, left) and Nbs1 phosphorylation (Figure 4C, right, compare lanes 2, 5 and 8). In contrast, addition of aphidicolin blocked DNA replication, but not Cdk2 activation or Nbs1 phosphorylation (Figure 4C, lanes 3 and 6). We conclude that Nbs1 is normally phosphorylated by CDKs after recruitment of the Mre11 complex to chromatin in S phase, but that this phosphorylation does not require ongoing DNA synthesis. Chromatin association of Nbs1, moreover, does not depend on Ser432 phosphorylation: in subcellular fractionation experiments, wild-type, S432A and S432D forms of Nbs1 had indistinguishable sensitivity to salt extraction from pelleted chromatin (Figure S2).

The timing of Nbs1-Ser432 phosphorylation coincided with the activation of Cdk2 (Figure 4A), but available inhibitors are incapable of distinguishing Cdk2- from Cdk1-dependent phosphorylations in wild-type cells. The *Cdk2^as/as^* cells [28] allowed us to ask if Nbs1-Ser432 is a preferred target of Cdk2 in vivo. We released *Cdk2^as/as^* or wild-type RPE-hTERT cells from G0, in the presence of either the allele-specific inhibitor 3-MB-PP1; or 6-benzylaminopurine (6-BAP), an adenine analog that corrects a cyclin-binding defect of Cdk2^as^ but does not inhibit its activity, and thereby rescues delayed G1 progression due to the hypomorphic *Cdk2^as^* mutation [28]. Neither drug affected Nbs1 phosphorylation in wild-type cells (Figure 4D and data not shown). In *Cdk2^as/as^* cells treated with 6-BAP or in wild-type cells treated with either drug, Nbs1-Ser432 phosphorylation appeared by 16 hr after release and increased over the next 8 hr. In contrast, 3-MB-PP1 treatment of *Cdk2^as/as^* cells diminished Nbs1 phosphorylation levels, relative to those in 6-BAP-treated cells (Figure 4D). Even in the presence of 3-MB-PP1, however, a phospho-Ser432 signal appeared in *Cdk2^as/as^* cells between 20 and 24 hr, suggesting that another kinase could phosphorylate this site in vivo, albeit with delayed kinetics. Therefore, Nbs1-Ser432 is phosphorylated preferentially by Cdk2, but might also be a target for another CDK, such as Cdk1, which is activated later in S phase [10], [11].

### Nbs1-Ser432 is required for normal resistance to IR

**Figure 5:**
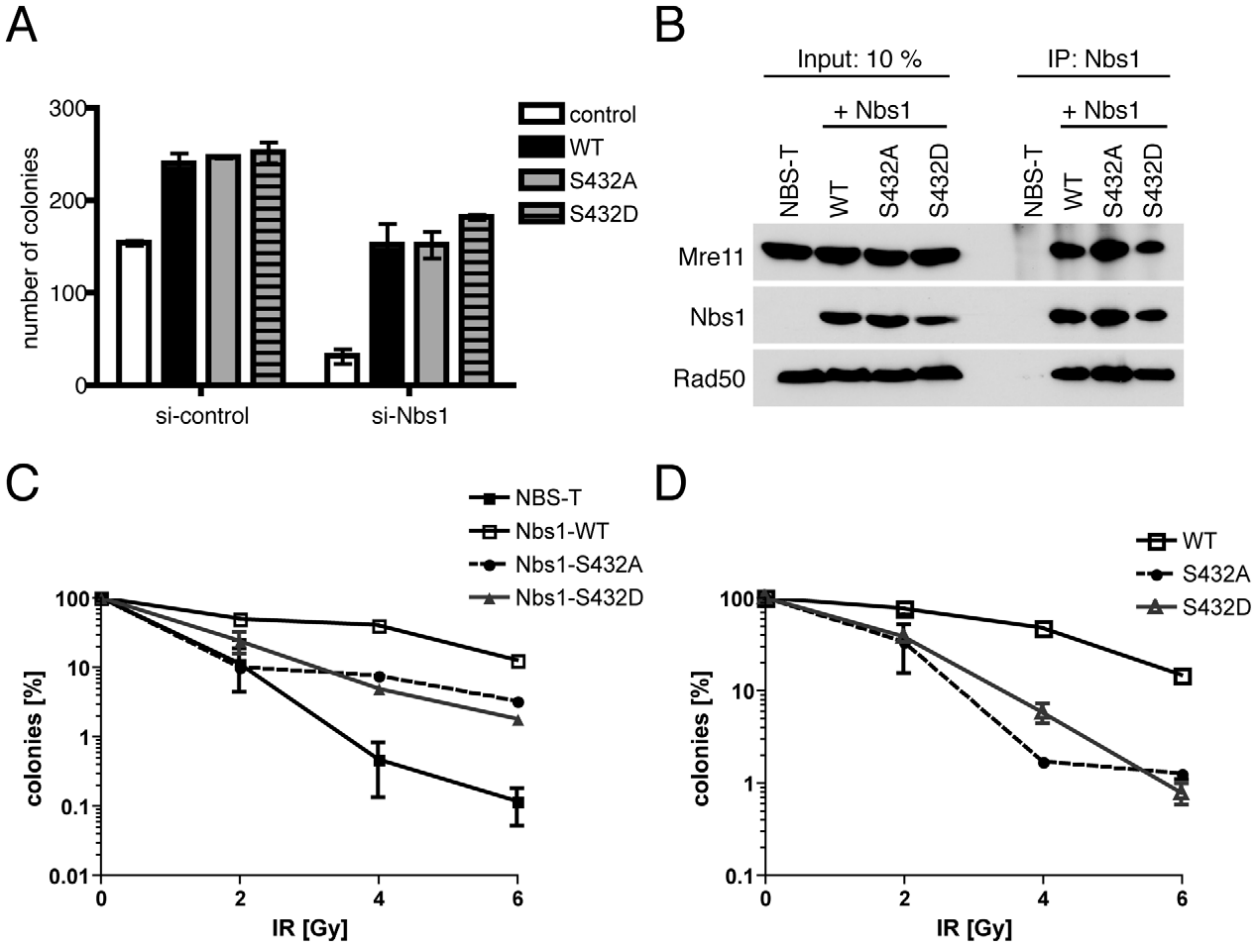
Nbs1-Ser432 is required for normal X-ray resistance but not viability. (A) Cells were treated with *Nbs1* or control siRNA and transiently transfected with empty vector or vectors encoding wild-type, S432A or S432D Nbs1 variants, as indicated, and tested for colony-forming ability. Error bars indicate +/− SD of duplicate measurements. (B) Nbs1 was immunoprecipitated from extracts of NBS-T cells, untransfected or stably expressing wild-type, S432A or S432D *Nbs1*, and probed for Nbs1, Rad50 and Mre11. (C) IR sensitivity of NBS-T cells expressing indicated Nbs1 isoforms. Cells were irradiated at indicated doses and tested for colony formation 14 d after irradiation. (D) IR sensitivity of cells from (C), transiently transfected with siRNA targeting *Nbs1*. (The parental NBS-T cells that express no full-length Nbs1 do not survive this treatment.) Values represent the means of duplicates +/− SD.

We next investigated possible requirements for Nbs1-Ser432 phosphorylation in vivo in NBS-T cells, which have impaired checkpoint signaling and reduced ability to repair DSBs by HR, leading to increased frequency of chromosomal aberrations and sensitivity to DNA-damaging agents (reviewed in [30]). These cells express the *Nbs1^657Δ5^* allele found in the majority of NBS patients, which encodes a 26 kDa amino-terminal fragment and a 70 kDa protein (p70) lacking the correct amino-terminus. The larger fragment retains Mre11-binding ability (and an intact Ser432 residue), and is likely to fulfill essential functions of Nbs1 in patients with NBS [45]. Depletion of residual Nbs1 by RNAi resulted in loss of colony forming ability (Figure 5A), indicating that p70 provides essential functions in NBS-T cells as well. Because the Nbs1 fragments are difficult to detect with available anti-Nbs1 antibodies [45], knockdown was verified by measuring RNA levels (Figure S3A). Loss of viability was due to depletion of *Nbs1* gene products rather than off-target effects, because it could be corrected by stable expression of RNAi-resistant, wild-type *Nbs1* (Figure 5A). The S432A and S432D versions were likewise capable of rescue, indicating that Ser432 phosphorylation is not required for survival. All three forms were expressed at equal levels, and co-immunoprecipitation of Mre11 and Rad50 revealed no effect of the mutations on complex formation (Figure 5B).

When irradiated in G2, NBS-T cells are defective in activating the G2/M checkpoint to restrain mitosis in the presence of DNA damage [46]. All three *Nbs1* variants complemented this defect to similar extents; the fraction of cells entering mitosis after exposure to IR was reduced (Figure S3B), and phosphorylation of the checkpoint kinase Chk2 was restored (Figure S3C). In fact, we observed low levels of Chk2 phosphorylation even in the absence of IR exposure in cells expressing Ser432 mutant forms of *Nbs1*, possibly suggesting accumulation of DNA damage under normal growth conditions. Nonetheless, we conclude that phosphorylation of Nbs1-Ser432 is not required for normal G2/M checkpoint function. Furthermore, Nbs1-Ser432 phosphorylation was not itself affected by DNA damage (Figure S3C).

We next asked if Nbs1-Ser432 was required for efficient recombinational repair, measured as the frequency of gene conversion of a split green fluorescent protein (GFP) reporter after introduction of a DSB by the endonuclease I-SceI [47]. NBS-T cells had low HR frequencies that were further reduced when endogenous Nbs1 fragments were depleted by RNAi (Figure S3D). Transient expression of RNAi-resistant Nbs1^WT^, Nbs1^S432A^ or Nbs1^S432D^ increased gene-conversion frequencies by similar amounts, indicating that phosphorylation of Nbs1-Ser432 is dispensable for HR. We also tested whether Nbs1-Ser432 phosphorylation was required for a specific function of the Mre11 complex in HR: resection from sites of DSBs to generate single-strand DNA (ssDNA) overhangs for strand invasion. We exposed NBS-T cells expressing no exogenous Nbs1, Nbs1^WT^, Nbs1^S432A^ or Nbs1^S432D^ to 4 Gy of IR and measured the number of cells positive for foci of the ssDNA-binding protein replication protein A (RPA) 1 and 6 hr later. There was no significant difference in RPA focus formation among the four genotypes (Figure S4), indicating that neither Nbs1-Ser432 nor full-length Nbs1 was required at the end-resection step. This also indicates that Nbs1 phosphorylation by CDKs is not required for recruitment of the Mre11 complex to damage foci, just as it did not appear to be needed for localization to bulk chromatin (Figure S2). We also measured Nbs1 focus formation directly and observed no difference between cells expressing wild-type or S432A alleles of *Nbs1* after exposure to 10 Gy IR (data not shown). Consistent with resection being normal in the absence of Nbs1-Ser432 phosphorylation, neither Nbs1-Ser432 substitution mutations (Figure S5A) nor inhibition of Cdk2^as^ in RPE-hTERT cells (Figure S5B) affected levels of CtIP, which promotes resection by interacting with the Mre11 complex [8], and which in turn is stabilized by its association with Mre11 [48].

Likewise, Nbs1-Ser432 phosphorylation does not seem to be required for the normal response to replication stress; Ser432 mutant *Nbs1* alleles fully complemented the hypersensitivity of NBS-T cells to chronic hydroxyurea (HU) exposure (Figure S6). In contrast, NBS-T cells stably complemented with S432A or S432D alleles of *Nbs1* were hypersensitive to killing by IR, compared to those complemented with wild-type *Nbs1*, although the parental NBS-T cells were more sensitive still (Figure 5C). We performed the same measurement after depleting residual Nbs1 by RNAi. (As expected because *Nbs1* is essential, after knockdown we recovered too few colonies of non-complemented NBS-T cells for reliable counting.) Both S432A- and S432D-expressing cells were more sensitive to killing by IR than were those expressing wild-type Nbs1 (Figure 5D). This suggests that phosphorylation of Nbs1-Ser432 contributes to the radioprotective effects of Cdk2 in human cells. Therefore, chemical genetics uncovered both a specific requirement for the activity of human Cdk2 in the response to DNA damage, and a specific target of Cdk2 within the DNA repair machinery.

## Discussion

### The cell-cycle machinery signals to the Mre11 complex

We have directly implicated a specific CDK-substrate interaction in the DNA damage response of human cells. By a chemical-genetic approach we identified Nbs1 as a target of Cdk2, and mapped the phosphorylation to a conserved CDK consensus recognition site. Both Cdk1 and Cdk2 phosphorylated Nbs1 with similar efficiency in vitro, and drugs that inhibit both CDKs—either directly (roscovitine or purvalanol A) or indirectly (3-MB-PP1 in *Cdk7^as/as^* cells)—diminished Nbs1-Ser432 phosphorylation in vivo more effectively than did inhibition of Cdk2 alone. These results are consistent with both Cdk2 and Cdk1 acting on Nbs1, in the temporal order in which they become activated during S phase [10], [11]. Nonetheless, Nbs1-Ser432 phosphorylation was delayed and diminished by selective Cdk2 inhibition during synchronous exit from G0, indicating a non-redundant function of Cdk2 in initiating Nbs1 phosphorylation. A preferential interaction between Cdk2 and the Mre11 complex is also suggested by the recent report that Cdk2/cyclin A, but not Cdk1, binds directly to a carboxyl-terminal motif in Mre11 [48].

Mre11, Rad50 and Nbs1 are essential, and participate in multiple pathways to protect genome integrity in mammalian cells (reviewed in [30], [49]). By depletion of endogenous Nbs1 fragments in NBS-T cells and complementation with mutant *Nbs1* alleles, we have shown here that: 1) the fragments can support essential and HR-related functions of the Mre11 complex in human somatic cells; and 2) Nbs1 phosphorylation by CDKs is not essential for viability, gene conversion or resistance to replicative stress, but contributes to normal IR-resistance. Because inhibition of Cdk2 also increased IR-sensitivity, the data suggest that Nbs1 functions downstream of Cdk2 in a DNA damage response pathway.

The Mre11 complex is recruited to chromatin during S phase [42], whereupon CDKs phosphorylate Nbs1 (this report), possibly to regulate Mre11-complex functions in response to DNA lesions incurred in replication [50]. Mutation of Ser432 to either Ala or Asp had similar effects on IR-sensitivity, suggesting that *de*phosphorylation of Nbs1 might also be important in vivo (or that Asp did not mimic phospho-Ser). Nbs1 is also recruited to human telomeres during late S or early G2 phase [51], [52], suggesting another, possibly cell cycle-regulated function of the Mre11 complex; we were unable, however, to detect consistent effects of *Nbs1* Ser432 mutations on telomere length in complemented NBS-T cells (unpublished observations).

### A specific requirement for Cdk2 in the DNA damage response

Taken together, our results support a role for Cdk2-mediated phosphorylation of Nbs1 in protecting genomic integrity. CDKs phosphorylate other proteins that work in concert with Nbs1. For example, the HR factor CtIP is phosphorylated during S/G2 and interacts directly with Nbs1 to promote repair focus formation in human cells [53]. Interaction of CtIP with BRCA1, another repair protein, depends on CtIP-Ser327 phosphorylation by a CDK [54]; and mutation of CtIP-Thr847, a second putative site of CDK phosphorylation, increases sensitivity to camptothecin [4]. Outstanding questions include: 1) Which aspects of Mre11-complex function depend on CDK activity? 2) Do Nbs1-Ser432 mutations exacerbate phenotypes due to loss of other CDK-mediated phosphorylations, e.g. on CtIP or BRCA2 [55]? 3) Might Cdk2 inhibition synergize with disruptions in other DNA damage response pathways to potentiate cell-killing by genotoxic agents?

To regulate DNA replication [56] and the G1/S transcriptional program [57], different CDKs phosphorylate multiple proteins within the relevant machineries. Specific CDK/cyclin pairs and phosphorylation sites can appear genetically redundant. Similar complexity is likely in DNA damage response pathways. Previous attempts to define functions of individual CDKs relied on: 1) relatively non-specific chemical inhibitors; or 2) gene disruptions or RNAi, which neither *allow* temporal control over enzymatic activity, nor *prohibit* non-physiologic compensation by other CDKs [15], [22]. Here, by a chemical-genetic strategy that preserved normal CDK-cyclin pairing [28], we uncovered non-redundant requirements for Cdk2 in human cells exposed to IR. Moreover, by manipulating Cdk2 activity selectively with small molecules, we could show that it is needed *after* damage occurs. This allowed us to exclude the possibility that increased IR-sensitivity was due to pre-existing defects in *Cdk2* mutant cells, and infer a requirement for Cdk2 to phosphorylate proteins de novo in response to DNA lesions [5]. The tools developed here should allow us to identify additional functions and targets of Cdk2 within DNA damage response pathways.

### A basis for Cdk2 specialization?

In budding yeast, checkpoint signaling arrests cell-cycle progression without CDK inhibition [58]. Cdk1 activity is required at multiple steps in HR, and several of its relevant targets have been identified (reviewed in [5], [6]). In mammalian cells, which rely on CDK inhibition for G2 arrest in response to DNA lesions, CDK substrates have nevertheless been identified in DNA damage repair [54], [55], [59]–[61] and checkpoint [62], [63] pathways. However, an exclusive, catalytic role for a specific CDK in protecting genome integrity had yet to be established. It was recently shown that *Cdk2^−/−^* human cells are defective in implementing a p53-independent G2/M checkpoint arrest [21]. This suggested a non-redundant role for Cdk2 protein but not necessarily for its catalytic activity. Cdk2 has a non-catalytic scaffold function that prevents premature assembly of Cdk1-cyclin complexes [28]; bypass of a checkpoint could therefore be due to ectopic activation of Cdk1 in *Cdk2^−/−^ p53^−/−^* cells.

**Figure 6:**
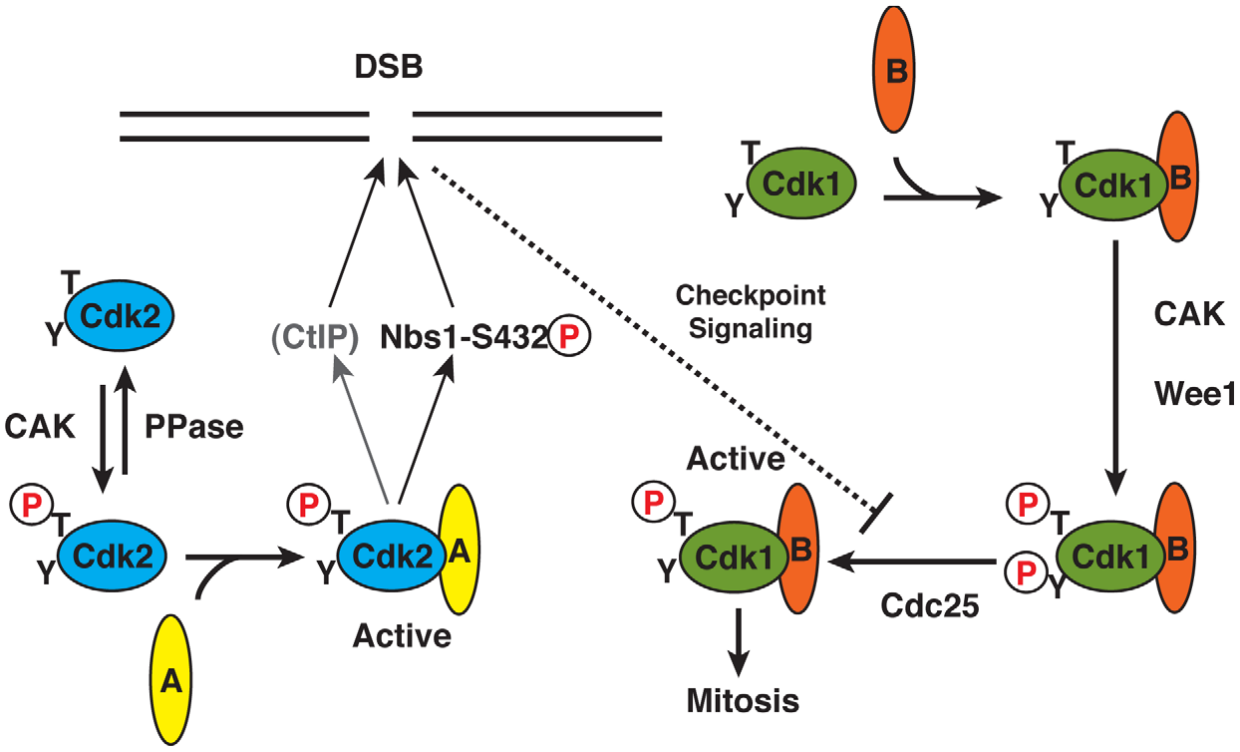
Specialized roles of Cdk2 in DNA damage response: a function of activation pathway insulation? We propose that a specific requirement for Cdk2 activity to protect cells from IR reflects its distinct mode of activation. Cdk2 is phosphorylated as a monomer by CAK, and then binds cyclin A to become active. Cdk1, in contrast, can only be phosphorylated by mammalian CAK in the presence of a cyclin (A or B), and only forms stable complexes with cyclins upon T-loop phosphorylation. This effectively couples Cdk1-activating phosphorylation to inhibitory phosphorylation by kinases such as Wee1 that also require a CDK/cyclin complex substrate [27], [66], and makes Cdk1 intrinsically more sensitive to restraint by DNA damage checkpoints. By evading that restraint, Cdk2 might take the lead role in phosphorylating Nbs1-Ser432 (and possibly other targets such as CtIP) early in S phase.

Here we have demonstrated a specific requirement for the catalytic activity of Cdk2 in survival of human cells exposed to IR—a DNA-damaging and checkpoint-activating treatment. This suggests that Cdk2 retains activity in this setting, perhaps by virtue of its distinctive mode of regulation (Figure 6). In contrast to Cdk1, which must interact with a cyclin in order to be phosphorylated by Cdk7, Cdk2 is phosphorylated predominantly as a monomer and then binds cyclin to become activated [10], [27]. Possibly as a consequence of this pathway insulation, Cdk2 is relatively refractory, during unperturbed cell cycles, to *inhibitory* phosphorylation [64]—the principal mechanism by which DNA structure checkpoints inactivate Cdk1 and restrain mitosis in mammalian cells [65]. We propose that the ability to evade inhibitory modification, and inability to trigger mitosis, are specific adaptations that permit Cdk2 to function efficiently and safely in the presence of DNA damage. In the future, it will be interesting to test whether Cdk2 activity is required for a p53-independent G2/M checkpoint pathway in *Cdk2^as/as^* cells, in which aberrant Cdk1-cyclin binding can be prevented. If so, targeting Cdk2 with specific inhibitors could be an attractive therapeutic strategy in human cancers with *p53* mutations.

## Materials and Methods

### Cell culture, extract preparation, and immunological methods

RPE-hTERT or HCT116 cells were grown in Dulbecco's modified Eagle medium (DMEM):F-12 or McCoy's 5A medium, respectively, supplemented with 10% fetal bovine serum (FBS). NBS-T cells were grown in DMEM with 10% FBS. Synchronization of RPE-hTERT cells was performed as described previously [28]. To detect Nbs1 in unfractionated extracts, cells were sonicated on ice with 2×5 s pulses of a 550 Sonic Dismembrator (Fisher Scientific). Chromatin fractionation was performed as described [42]. Immunoblots and immunoprecipitations were carried out as described [33] with the following antibodies: anti-Cdk2 (M2) and anti-cyclin A (H432) from Santa-Cruz Biotechnology; anti-Cdk1 (POH1), anti-P-T160-Cdk2, anti-P-T161-Cdk1 and anti-P-T68-Chk2 from Cell Signaling Technologies; anti-Chk2 (clone 7) from Upstate; and anti-Mre11, anti-Rad50, and anti-Nbs1 from Novus Biologicals. For detection of phosphorylated Nbs1 we used anti-P-S432-Nbs1 from Abcam: either total Nbs1 was immunoprecipitated from extracts and probed for phospho-Ser432, or extracts were electrophoresed in 3–8% tris-acetate gradient gels (NuPAGE Novex, Invitrogen).

### Kinase assays

The indicated CDK/cyclin complexes were reconstituted in vitro from purified subunits expressed in baculovirus-infected insect cells (Cdk1, Cdk2^as^, Cdk2^WT^, cyclin A, cyclin B) and activated by CAK; or co-expressed and purified as complexes (Cdk7/cyclin H to which we added Mat1 purified separately, and Cdk9/cyclin T1). GST-Nbs1(397-742) was expressed in bacteria. Kinase reactions were carried out on histone H1, GST-Nbs1(397-742) or Cdk2 substrates. Substrates (1.0–8.5 µg) were incubated for 10–15 min at 22°C with CDK complexes in 20 µl kinase buffer (10 mM HEPES, pH 7.4, 150 mM NaCl, 10 mM MgCl_2_, 200 µM ATP) with or without 10 µCi [γ-^32^P]ATP. Reaction products were analyzed by 10% SDS–PAGE followed by autoradiography or immunoblot analysis.

### Chemical-genetic methods

Treatment of cells with 3-MB-PP1 or 6-BAP was performed as previously described [28]. To label substrates of Cdk2, 100 µg of whole-cell extract protein was incubated for 15 min at room temperature with 140 ng Cdk2^as^-His/cyclin A complex in a 60-µl reaction containing an ATP regenerating system (25 mM Hepes, pH 7.4, 10 mM NaCl, 2 mM MgCl_2_, 1 mM ATP, 40 mM creatine phosphate, 0.2 mg/ml creatine phosphokinase) and 5 µCi [γ-^32^P]*N* ^6^(benzyl)-ATP, as described previously [26], [32], [33]. Labeling was stopped by addition of 2× sample buffer. Phosphorylated proteins were separated by sodium dodecylsulfate-polyacrylamide gel electrophoresis (SDS-PAGE) and detected by autoradiography. Labeling reactions were scaled up to 400 µg extract protein for immunoprecipitation. To express Nbs1 in vitro, we used TNT® Coupled Reticulocyte Lysate kit from Promega, and performed analog-selective labeling, as previously described for mapping of sites phosphorylated by Cdk7^as^ [32]. 3-MB-PP1 was dissolved in dimethylsulfoxide (DMSO) and used at 0–10 µM.

### Transfection and retroviral infection of NBS-T cells

Small interfering RNA (siRNA) homologous to human *Nbs1*, 5′-CCAACAAGGUUAUAUGAAU-3′, and a negative control duplex of random sequence, 5′-GGUGGACGGCAAGUUUGCU-3′, were synthesized by Dharmacon Research. For complementation of NBS-T cells, we used expression plasmids pcDNA3-Nbs1myc and pCLN-Nbs1. Phosphorylation-site mutations (S432A or S432D) were introduced by site-directed mutagenesis. Logarithmically growing cells were transfected with siRNA and/or expression plasmids using Lipofectamine 2000 (Invitrogen) following manufacturer's instructions. For retrovirus production we used a Phoenix Retroviral Expression System.

### Colony formation assay

To determine sensitivity of cells to different agents, 500–40,000 cells (depending on the treatment) were plated in 10-cm^2^ dishes. Colonies were counted 14 d later after staining with crystal violet.

Additional methods used to generate data in Supporting Figures are described in Text S1.

## Supporting Information

Figure S1 Nbs1 protein levels and labeling by Cdk2^as^ in extracts of different human cell lines. Whole-cell extracts of indicated cell lines (“LCL2985wt” is a lymphoblastoid cell line; “NBS-T+Nbs1” is an NBS-T cell line stably complemented with wild-type *Nbs1*) were labeled with recombinant Cdk2^as^/cyclin A and [γ-^32^P]*N6*-(benzyl)-ATP, followed by anti-Nbs1 immunoprecipitation and immunoblot analysis of Nbs1 (top) or autoradiography (second from top). The same extracts were also probed directly by immunoblot (without immunoprecipitation) for Nbs1 (third from top) or the loading control glyceraldehyde 3-phosphate dehydrogenase (GAPDH; bottom).
(TIF)

Figure S2 Ser432 is not required for Nbs1 localization to chromatin. Fractionation of NBS-T cells stably expressing wild-type, S432A, or S432D Nbs1 was performed as in Figure 4B. Isolated nuclei were extracted with different concentrations of NaCl, as indicated, and Nbs1 protein levels were measured in soluble fractions derived from equal numbers of cell-equivalents.
(TIF)

Figure S3 Phenotypic characterization of Nbs1-Ser432 phosphorylation. (A) Total RNA of NBS-T cells treated either with control siRNA (si-control) or siRNA targeting endogenous *Nbs1* mRNA (si-Nbs1) was used as template in reverse transcriptase polymerase chain reaction (RT-PCR) and subsequent PCR with *Nbs1*- or *Cdk2*-specific primers to estimate transcript abundance. Treatment with siRNA specific to *Nbs1* led to a reduction in *Nbs1* mRNA levels whereas *Cdk2* mRNA levels were not affected. (B) G2/M checkpoint assay of Nbs1-deficient NBS-T cells or NBS-T cells stably complemented with indicated alleles of *Nbs1*. Mitotic fraction was quantified by phosphorylated histone H3 staining 1 hr after X-irradiation with 10 Gy. Error bars denote +/− SD from duplicate measurements of two independent clones of each genotype. (C) Nbs1-Ser432 and Chk2-Thr68 phosphorylation of cells in (B) after X-irradiation. Note that Chk2 Thr-68 phosphorylation occurs at low levels in cells expressing Nbs1^S432A^ even in the absence of X-rays. (D) NBS-T/DR-GFP cells were treated with siRNA targeting *Nbs1* or control siRNA and transiently complemented with empty vector or wild-type, S432A or S432D alleles of *Nbs1*, as indicated, and tested for gene-conversion frequency after I-SceI expression. Numbers of GFP-positive cells are expressed as percentages of the value in cells. complemented with wild-type *Nbs1* (defined as 100%); error bars denote +/− SD of three independent experiments.
(TIF)

Figure S4 Nbs1-Ser432 is not required for DNA damage focus formation. RPA focus formation was measured in parental NBS-T cells or NBS-T cells stably expressing wild-type, S432A or S432D alleles of *Nbs1*. Cells were γ-irradiated with 4 Gy and collected for RPA immunostaining at 1 and 6 hr post-irradiation. RPA focus-positive cells were counted from more than 130 randomly chosen cells for each cell line.
(TIF)

Figure S5 CtIP levels are unaffected by Nbs1 Ser432 mutation or Cdk2 inhibition. (A) CtIP protein levels were measured by immunoblotting of NBS-T cells stably expressing wild-type, S432A, or S432D alleles of *Nbs1*. (B) Wild-type or *Cdk2^as/as^* RPE-hTERT were treated with DMSO, 0.5 µM 6-BAP or 10 µM 3-MB-PP1 for 20 hr and tested for CtIP expression by immunoblotting.
(TIF)

Figure S6 Determination of HU sensitivity. NBS-T cells expressing either wild-type, S432A or S432D alleles of *Nbs1* were treated with the indicated doses of HU. Medium was changed 24 hr after start of treatment and cells were tested for colony formation after 14 d. Values represent the means of duplicates +/− SD.
(TIF)

Text S1 Methods used to generate data in Figures S1, S2, S3, S4, S5, S6 are described in Text S1.
(DOC)
