# Supplementary figures and images for: Chemical Genetics Reveals a Specific Requirement for Cdk2 Activity in the DNA Damage Response and Identifies Nbs1 as a Cdk2 Substrate in Human Cells
Source: PLoS Genet. 2012 Aug 23;8(8):e1002935. doi: 10.1371/journal.pgen.1002935 (PMC3426557; doi:10.1371/journal.pgen.1002935)

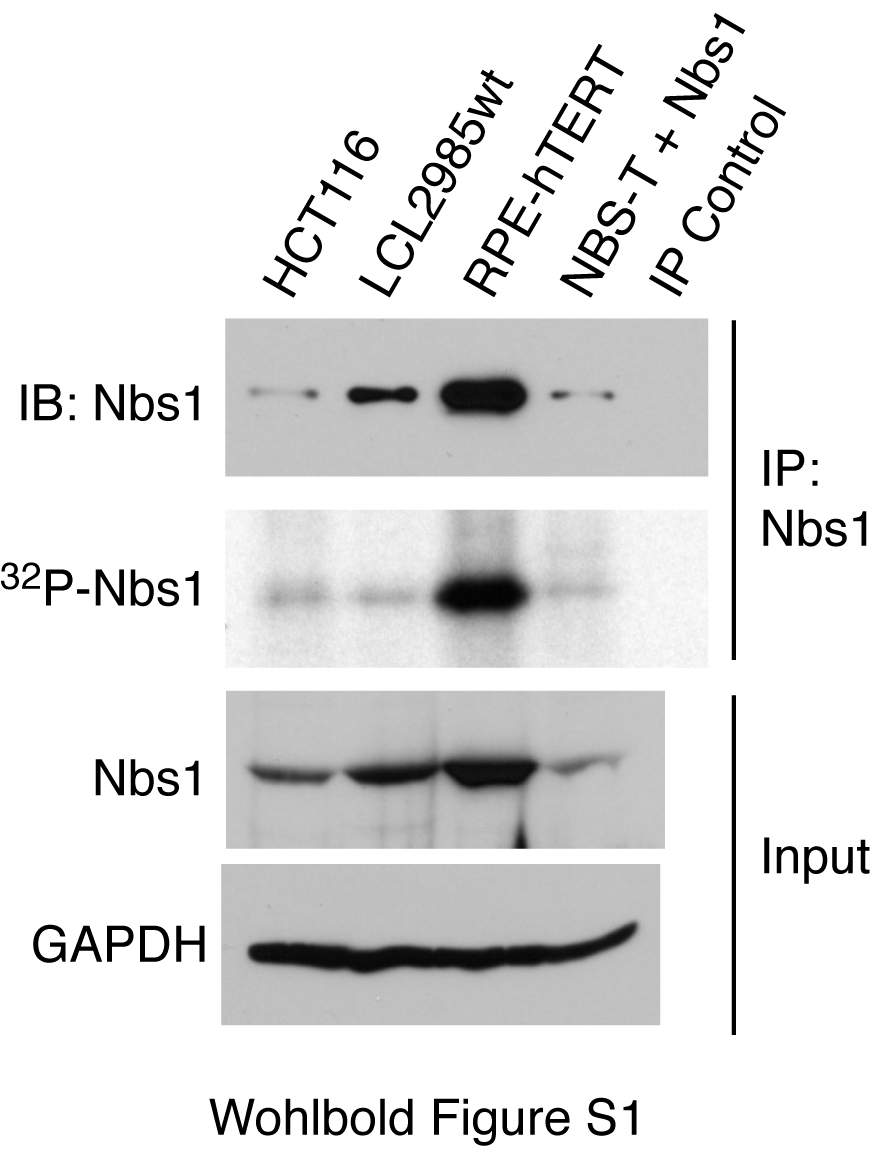

Supplement: Figure S1 — Nbs1 protein levels and labeling by Cdk2as in extracts of different human cell lines. Whole-cell extracts of indicated cell lines (“LCL2985wt” is a lymphoblastoid cell line; “NBS-T+Nbs1” is an NBS-T cell line stably complemented with wild-type Nbs1) were labeled with recombinant Cdk2as/cyclin A and [γ-32P]N6-(benzyl)-ATP, followed by anti-Nbs1 immunoprecipitation and immunoblot analysis of Nbs1 (top) or autoradiography (second from top). The same extracts were also probed directly by immunoblot (without immunoprecipitation) for Nbs1 (third from top) or the loading control glyceraldehyde 3-phosphate dehydrogenase (GAPDH; bottom). (TIF) [file pgen.1002935.s001.tif]

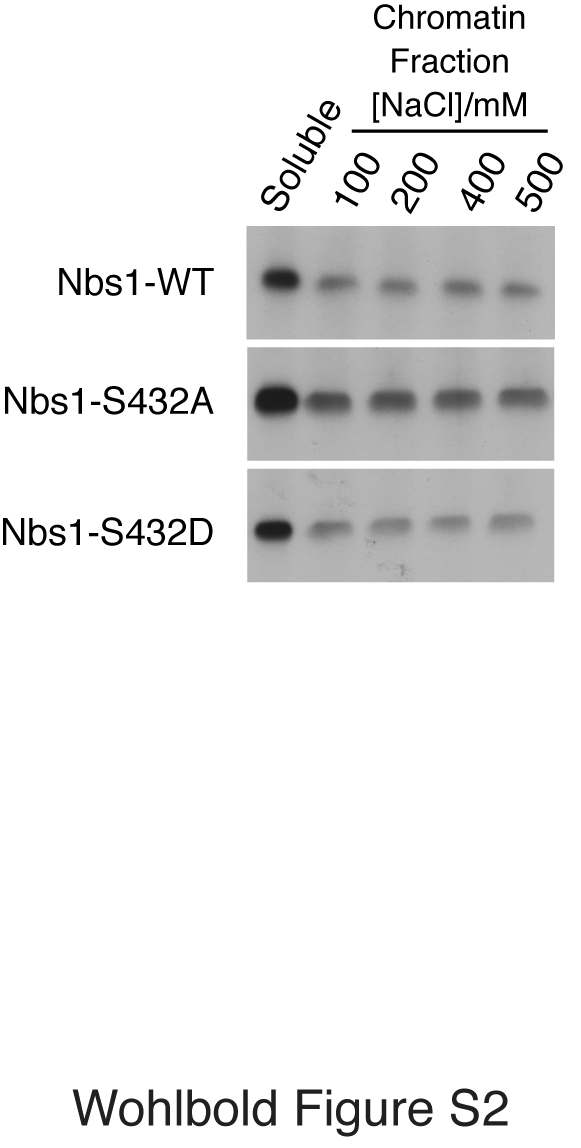

Supplement: Figure S2 — Ser432 is not required for Nbs1 localization to chromatin. Fractionation of NBS-T cells stably expressing wild-type, S432A, or S432D Nbs1 was performed as in Figure 4B. Isolated nuclei were extracted with different concentrations of NaCl, as indicated, and Nbs1 protein levels were measured in soluble fractions derived from equal numbers of cell-equivalents. (TIF) [file pgen.1002935.s002.tif]

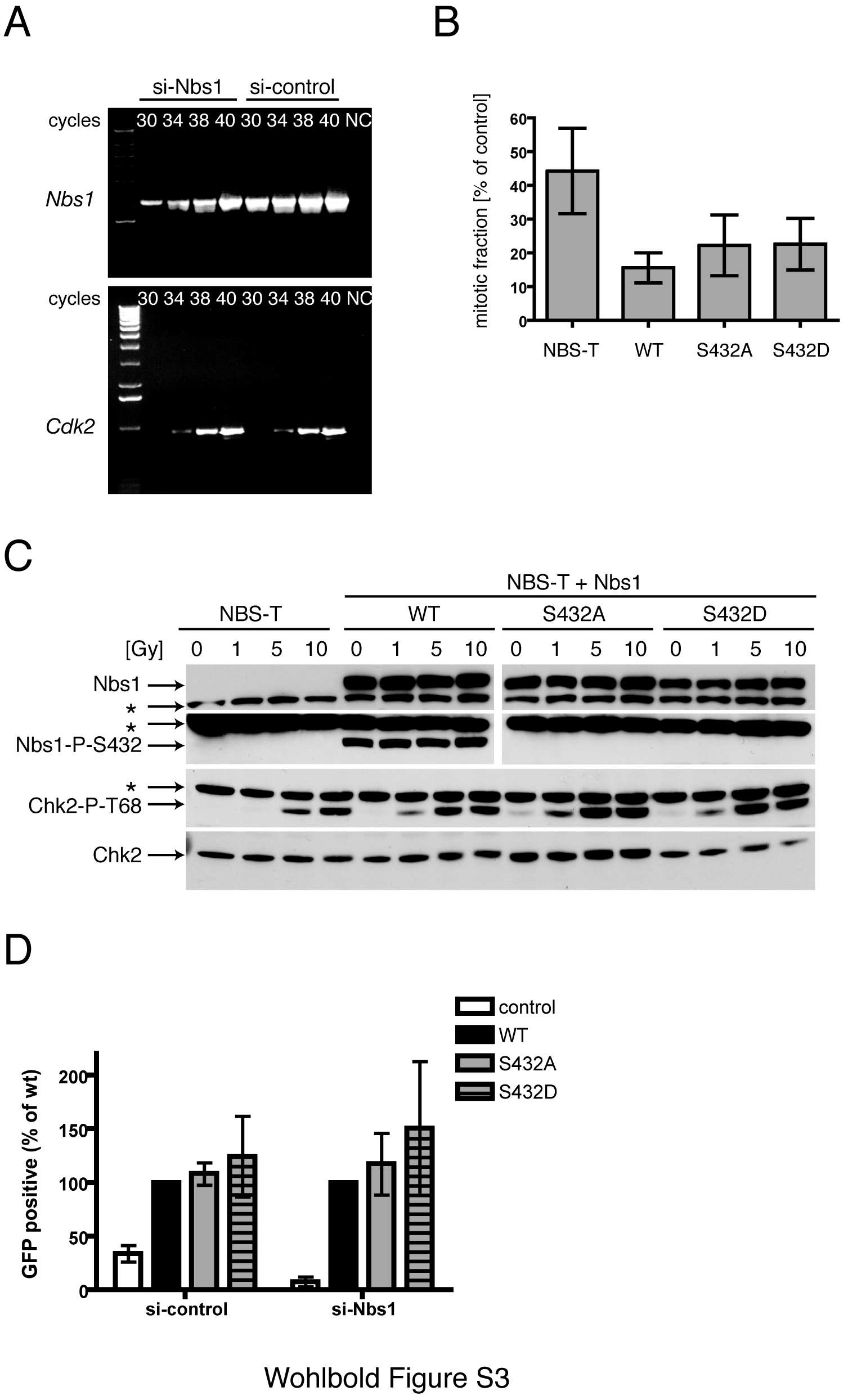

Supplement: Figure S3 — Phenotypic characterization of Nbs1-Ser432 phosphorylation. (A) Total RNA of NBS-T cells treated either with control siRNA (si-control) or siRNA targeting endogenous Nbs1 mRNA (si-Nbs1) was used as template in reverse transcriptase polymerase chain reaction (RT-PCR) and subsequent PCR with Nbs1- or Cdk2-specific primers to estimate transcript abundance. Treatment with siRNA specific to Nbs1 led to a reduction in Nbs1 mRNA levels whereas Cdk2 mRNA levels were not affected. (B) G2/M checkpoint assay of Nbs1-deficient NBS-T cells or NBS-T cells stably complemented with indicated alleles of Nbs1. Mitotic fraction was quantified by phosphorylated histone H3 staining 1 hr after X-irradiation with 10 Gy. Error bars denote +/− SD from duplicate measurements of two independent clones of each genotype. (C) Nbs1-Ser432 and Chk2-Thr68 phosphorylation of cells in (B) after X-irradiation. Note that Chk2 Thr-68 phosphorylation occurs at low levels in cells expressing Nbs1S432A even in the absence of X-rays. (D) NBS-T/DR-GFP cells were treated with siRNA targeting Nbs1 or control siRNA and transiently complemented with empty vector or wild-type, S432A or S432D alleles of Nbs1, as indicated, and tested for gene-conversion frequency after I-SceI expression. Numbers of GFP-positive cells are expressed as percentages of the value in cells. complemented with wild-type Nbs1 (defined as 100%); error bars denote +/− SD of three independent experiments. (TIF) [file pgen.1002935.s003.tif]

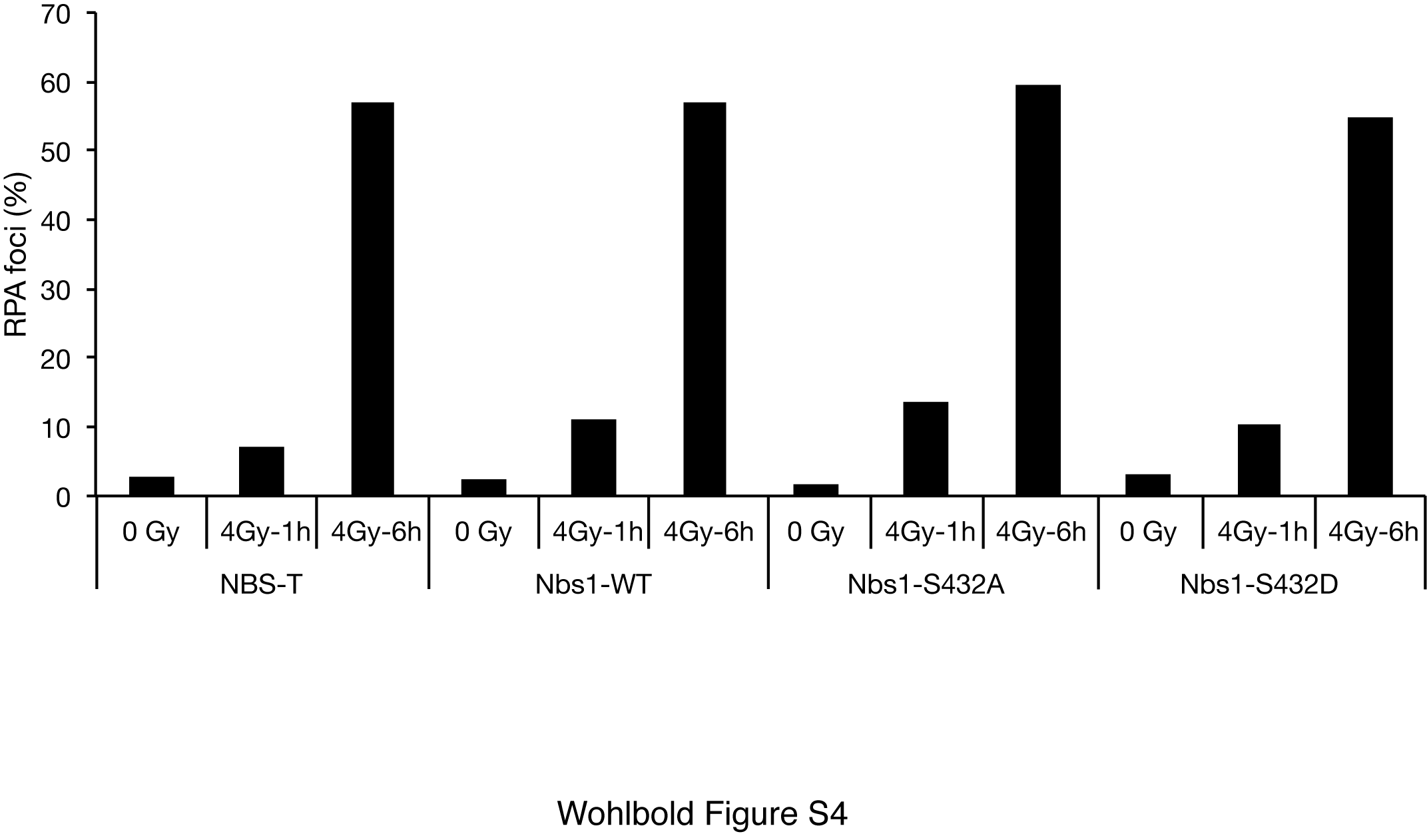

Supplement: Figure S4 — Nbs1-Ser432 is not required for DNA damage focus formation. RPA focus formation was measured in parental NBS-T cells or NBS-T cells stably expressing wild-type, S432A or S432D alleles of Nbs1. Cells were γ-irradiated with 4 Gy and collected for RPA immunostaining at 1 and 6 hr post-irradiation. RPA focus-positive cells were counted from more than 130 randomly chosen cells for each cell line. (TIF) [file pgen.1002935.s004.tif]

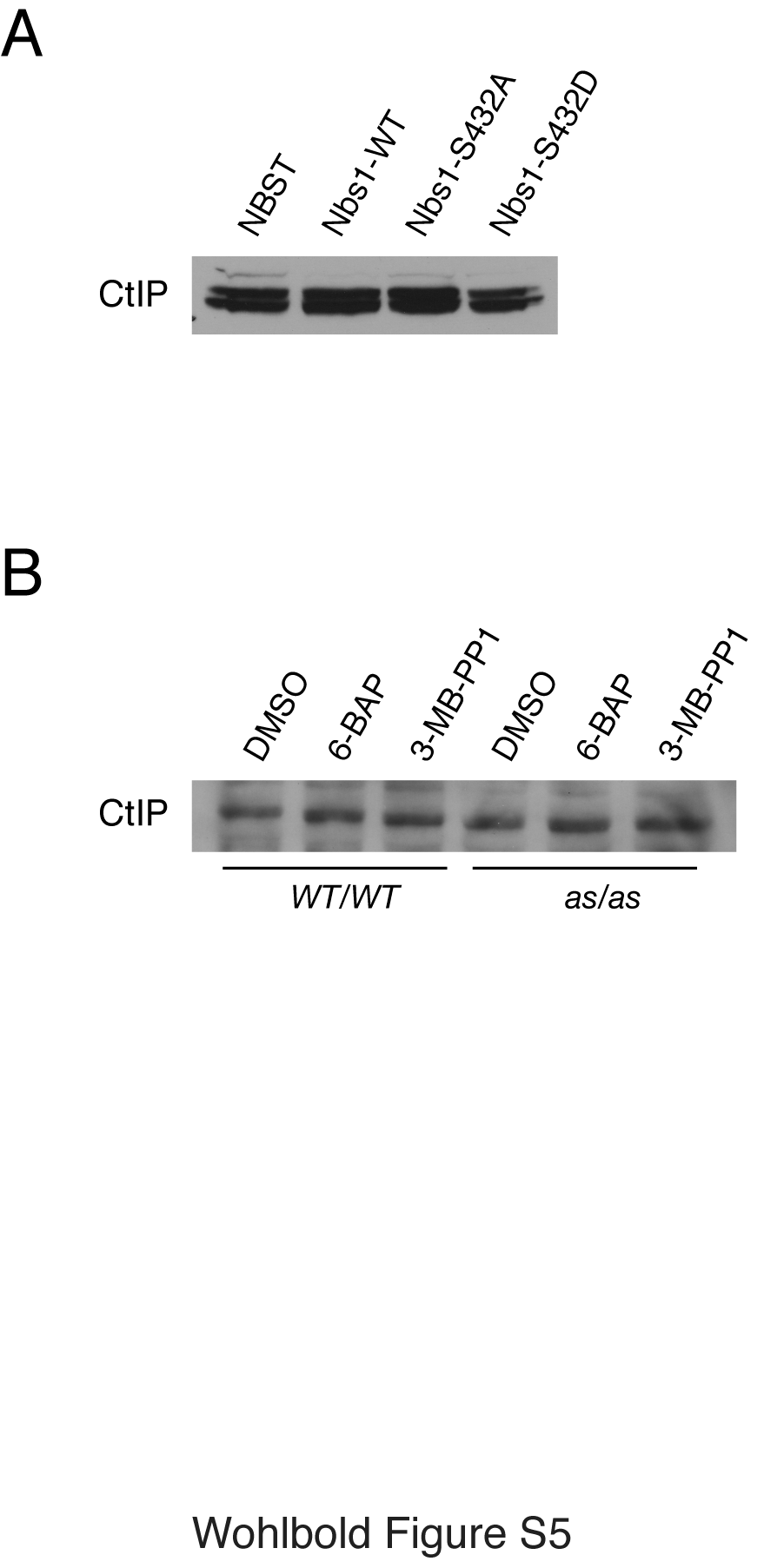

Supplement: Figure S5 — CtIP levels are unaffected by Nbs1 Ser432 mutation or Cdk2 inhibition. (A) CtIP protein levels were measured by immunoblotting of NBS-T cells stably expressing wild-type, S432A, or S432D alleles of Nbs1. (B) Wild-type or Cdk2as/as RPE-hTERT were treated with DMSO, 0.5 µM 6-BAP or 10 µM 3-MB-PP1 for 20 hr and tested for CtIP expression by immunoblotting. (TIF) [file pgen.1002935.s005.tif]

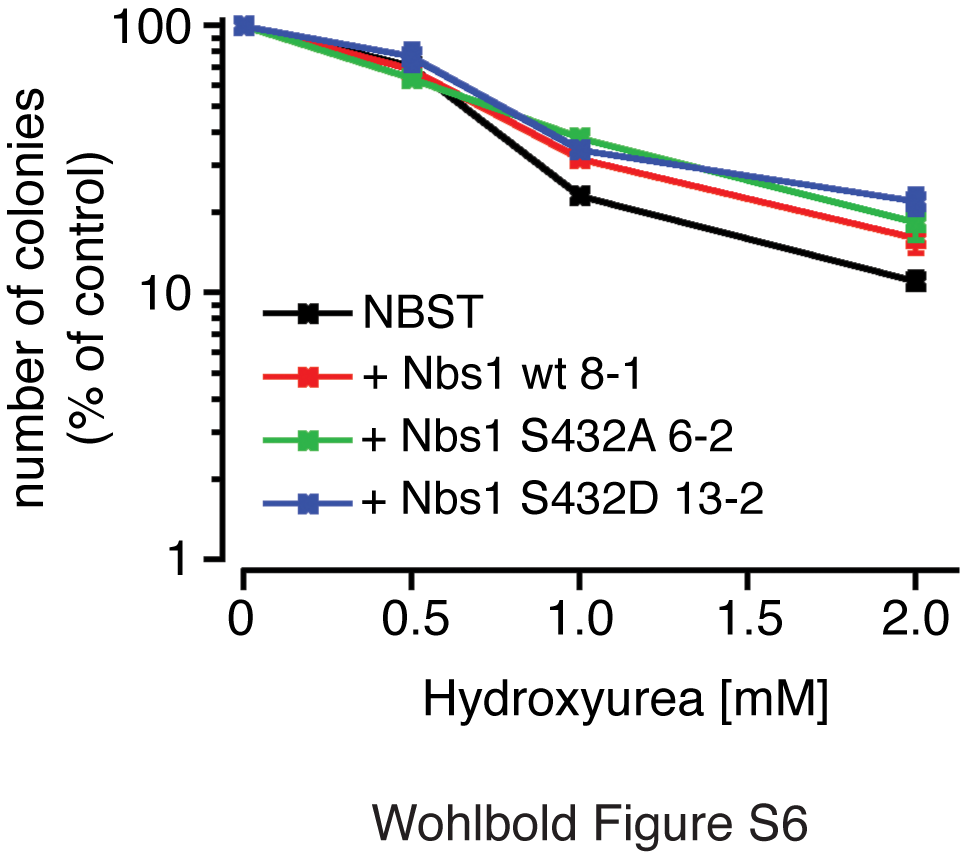

Supplement: Figure S6 — Determination of HU sensitivity. NBS-T cells expressing either wild-type, S432A or S432D alleles of Nbs1 were treated with the indicated doses of HU. Medium was changed 24 hr after start of treatment and cells were tested for colony formation after 14 d. Values represent the means of duplicates +/− SD. (TIF) [file pgen.1002935.s006.tif]
