# Supplementary material for: Chemical Genetics Reveals a Specific Requirement for Cdk2 Activity in the DNA Damage Response and Identifies Nbs1 as a Cdk2 Substrate in Human Cells
Source: PLoS Genet. 2012 Aug 23;8(8):e1002935. doi: 10.1371/journal.pgen.1002935 (PMC3426557; doi:10.1371/journal.pgen.1002935)
Supplement: Text S1 — Methods used to generate data in Figures S1, S2, S3, S4, S5, S6 are described in Text S1. (DOC) [file pgen.1002935.s007.doc]

**Supporting Information for:**

**Chemical genetics in human cells reveals a specific requirement for Cdk2 activity in the DNA damage response and identifies Nbs1 as a Cdk2 substrate**

**Lara Wohlbold *et al.***

**Supporting Materials and Methods**

**Nbs1 extractability assay**

Subcellular fractionation of NBS-T cells, or NBS-T cells stably expressing wild-type, S432A, or S432D alleles of *Nbs1*, into soluble and nuclear fractions was performed as described [1]. Isolated nuclei were extracted with different concentrations of NaCl (100, 200, 400, or 500 mM). The extracts were normalized to each other on the basis of cell number-equivalence and Nbs1 levels were measured by immunoblotting.

**G2/M-checkpoint assay**

Measurement of mitotic cell population after X-irradiation was performed as described previously [2].

**RPA focus formation assay**

NBS-T cells or NBS-T cells stably expressing wild-type, S432A or S432D mutant alleles of *Nbs1* were grown on glass coverslips and γ-irradiated with 4 Gy. Cells were pre-extracted with phosphate-buffered saline (PBS) containing 0.5% Triton X-100 at room temperature for 5 min and fixed for 10 min with PBS containing 2% paraformaldehyde. Cells were washed in PBS containing 0.1% Tween 20 (PBS-T) and blocked for 1 hr in PBS-T containing 2% fetal calf serum. Cells were incubated for 1 hr at room temperature with RPA antibodies (Abcam, 9H8) in PBS-T containing 2% fetal calf serum (1:1000 dilution). The cells were then washed three times for 5 min each in PBS-T and incubated for 1 hr at room temperature with anti-mouse Alexa-Fluor-488 (1:1000 dilution in PBS-T containing 2% fetal calf serum). Cells were washed three times for 10 min each with PBS-T containing 2% fetal calf serum and mounted with ProLong® Gold Antifade Reagent with DAPI (Molecular Probe).

**HR assay**

Stable integrants containing tandem copies of non-functional mutant GFP genes, one of which contains an I-SceI site, were obtained by transfecting NBS-T cells with linearized hprtDR-GFPhygro, selecting for hygromycin-resitance, and confirming genomic integration by Southern blotting [3]. NBS-T/DR-GFP cells were next infected with retrovirus carrying either Nbs1-WT, Nbs1-S432A or Nbs1-S432D cDNA and a *neoR* marker. To measure the repair of an I-SceI-induced DSB, infected cells were selected in medium containing 400 µg/ml G418 for 2-3 weeks before transient transfection with an I-*Sce*I expression vector. GFP-positive cells were quantified 2 days after I-SceI transfection by flow cytometry on a Becton Dickinson FACScan.

**HU sensitivity assay**

To determine the sensitivity of NBS-T cells expressing either wild-type, S432A or S432D alleles of *Nbs1* to hydroxyurea (HU), between 500 and 40,000 cells (depending on HU dose) were seeded in 10-cm^2^ dishes and treated with the indicated doses of HU one day after seeding. After a 24-hr HU treatment, cells were washed with PBS and fresh medium was added. Colonies were counted 14 d later after staining with crystal violet.

**Supporting Figures**

**Figure S1 Nbs1 protein levels and labeling by Cdk2^as^ in extracts of different human cell lines**

Whole-cell extracts of indicated cell lines (“LCL2985wt” is a lymphoblastoid cell line; “NBS-T + Nbs1” is an NBS-T cell line stably complemented with wild-type *Nbs1*) were labeled with recombinant Cdk2^as^/cyclin A and [γ-^32^P]*N6*-(benzyl)-ATP, followed by anti-Nbs1 immunoprecipitation and immunoblot analysis of Nbs1 (top) or autoradiography (second from top). The same extracts were also probed directly by immunoblot (without immunoprecipitation) for Nbs1 (third from top) or the loading control glyceraldehyde 3-phosphate dehydrogenase (GAPDH; bottom).

**Figure S2 Ser432 is not required for Nbs1 localization to chromatin**

Fractionation of NBS-T cells stably expressing wild-type, S432A, or S432D Nbs1 was performed as in Figure 4B. Isolated nuclei were extracted with different concentrations of NaCl, as indicated, and Nbs1 protein levels were measured in soluble fractions derived from equal numbers of cell-equivalents.

**Figure S3** **Phenotypic characterization of Nbs1-Ser432 phosphorylation**

(A) Total RNA of NBS-T cells treated either with control siRNA (si-control) or siRNA targeting endogenous *Nbs1* mRNA (si-Nbs1) was used as template in reverse transcriptase polymerase chain reaction (RT-PCR) and subsequent PCR with *Nbs1*- or *Cdk2*-specific primers to estimate transcript abundance. Treatment with siRNA specific to *Nbs1* led to a reduction in *Nbs1* mRNA levels whereas *Cdk2* mRNA levels were not affected.

(B) G2/M checkpoint assay of Nbs1-deficient NBS-T cells or NBS-T cells stably complemented with indicated alleles of *Nbs1*. Mitotic fraction was quantified by phosphorylated histone H3 staining 1 hr after X-irradiation with 10 Gy. Error bars denote +/- SD from duplicate measurements of two independent clones of each genotype.

(C) Nbs1-Ser432 and Chk2-Thr68 phosphorylation of cells in (B) after X-irradiation. Note that Chk2 Thr-68 phosphorylation occurs at low levels in cells expressing Nbs1^S432A^ even in the absence of X-rays.

(D) NBS-T/DR-GFP cells were treated with siRNA targeting *Nbs1* or control siRNA and transiently complemented with empty vector or wild-type, S432A or S432D alleles of *Nbs1*, as indicated, and tested for gene-conversion frequency after I-SceI expression. Numbers of GFP-positive cells are expressed as percentages of the value in cells. complemented with wild-type *Nbs1* (defined as 100%); error bars denote +/- SD of three independent experiments.

**Figure S4 Nbs1-Ser432 is not required for DNA damage focus formation**

RPA focus formation was measured in parental NBS-T cells or NBS-T cells stably expressing wild-type, S432A or S432D alleles of *Nbs1*. Cells were γ-irradiated with 4 Gy and collected for RPA immunostaining at 1 and 6 hr post-irradiation. RPA focus-positive cells were counted from more than 130 randomly chosen cells for each cell line.

**Figure S5 CtIP levels are unaffected by Nbs1 Ser432 mutation or Cdk2 inhibition**

(A) CtIP protein levels were measured by immunoblotting of NBS-T cells stably expressing wild-type, S432A, or S432D alleles of *Nbs1*.

(B) Wild-type or *Cdk2^as/as^* RPE-hTERT were treated with DMSO, 0.5 μM 6-BAP or 10 μM 3-MB-PP1 for 20 hr and tested for CtIP expression by immunoblotting.

**Figure S6** **Determination of HU sensitivity**

NBS-T cells expressing either wild-type, S432A or S432D alleles of *Nbs1* were treated with the indicated doses of HU. Medium was changed 24 hr after start of treatment and cells were tested for colony formation after 14 d. Values represent the means of duplicates +/- SD.

**Supporting References**

1. Mirzoeva OK, Petrini JH (2003) DNA replication-dependent nuclear dynamics of the Mre11 complex. Mol Cancer Res 1: 207-218.

2. Theunissen JW, Petrini JH (2006) Methods for studying the cellular response to DNA damage: influence of the Mre11 complex on chromosome metabolism. Methods Enzymol 409: 251-284.

3. Nakanishi K, Yang YG, Pierce AJ, Taniguchi T, Digweed M, et al. (2005) Human Fanconi anemia monoubiquitination pathway promotes homologous DNA repair. Proc Natl Acad Sci U S A 102: 1110-1115.
